# Correction: Constructing Benchmark Test Sets for Biological Sequence Analysis Using Independent Set Algorithms

**DOI:** 10.1371/journal.pcbi.1010971

**Published:** 2023-03-08

**Authors:** Samantha Petti, Sean R Eddy

The description of Algorithm 2 at the bottom of page 4 is incorrect. The corrected pseudocode reads like:

**If** *r* < 1/2 **then**

    **if** *v is not adjacent to any vertex in T*, **add** *v to S*

    **else if** *v is not adjacent to any vertex in S*, **add** *v to T*

else

    **if** *v is not adjacent to any vertex in S*, **add** *v to T*

    **else if** *v is not adjacent to any vertex in T*, **add** *v to S*
