# Supplementary figures and images for: Constructing benchmark test sets for biological sequence analysis using independent set algorithms
Source: PLoS Comput Biol. 2022 Mar 7;18(3):e1009492. doi: 10.1371/journal.pcbi.1009492 (PMC8929697; doi:10.1371/journal.pcbi.1009492)

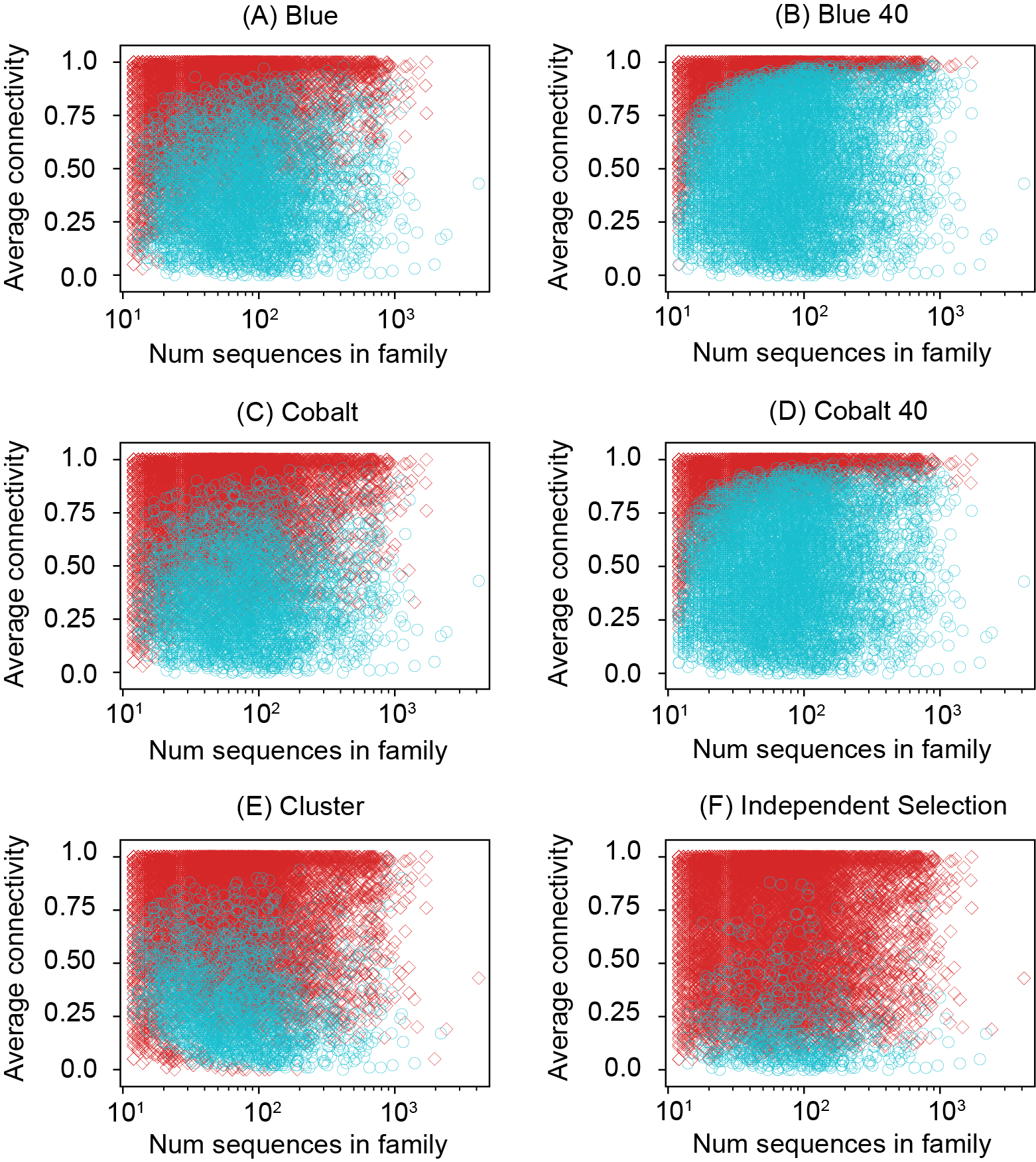

Supplement: S1 Fig — Each marker represents a family in Pfam. The connectivity of a sequence is the fraction of other sequences in the seed family with at least 25% pairwise identity. Families successfully split into a training set of size at least 10 and a test set of size at least 2 are marked by a cyan circle, whereas families that were not split are marked by a red diamond. In (B) and (D) the cyan circle represents at least one successful split among 40 independent runs. (TIF) [file pcbi.1009492.s001.tif]

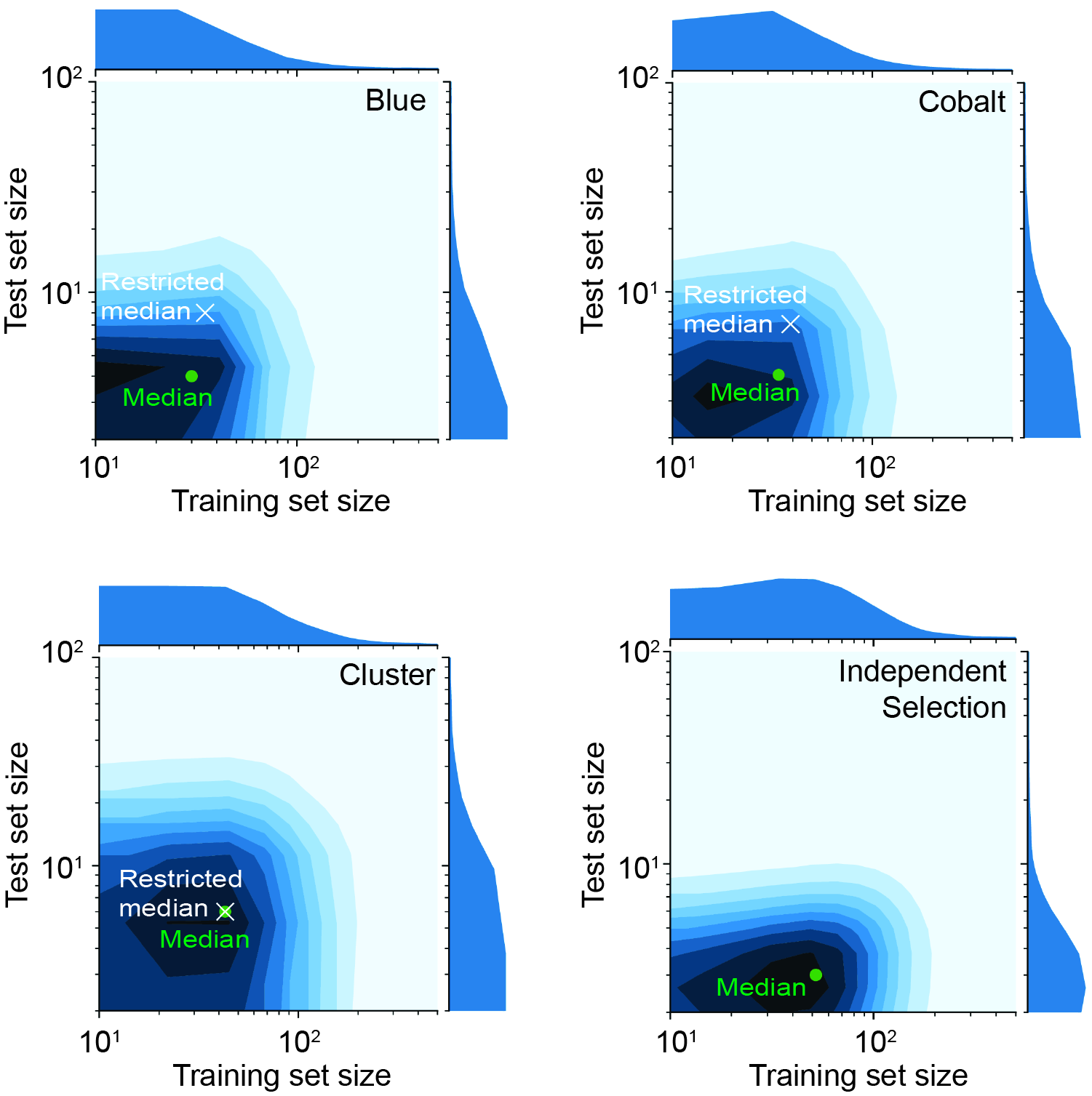

Supplement: S2 Fig — The two-dimensional normalized histograms illustrate the distribution of training and test set sizes produced by the algorithms among results with at least 10 and 2 training and test sequences respectively. In each plot, the x-coordinate and y-coordinates of the green circle represent the median training and median test set sizes respectively. The white X is placed at the median training and test set sizes among the 2363 families that were successfully split by Blue, Cobalt, and Cluster. (TIF) [file pcbi.1009492.s002.tif]
