# Supplementary material for: Constructing benchmark test sets for biological sequence analysis using independent set algorithms
Source: PLoS Comput Biol. 2022 Mar 7;18(3):e1009492. doi: 10.1371/journal.pcbi.1009492 (PMC8929697; doi:10.1371/journal.pcbi.1009492)
Supplement: Attachment — Submitted filename: response-to-reviews.pdf [file pcbi.1009492.s004.pdf]

3 January 2022

Maricel G Kann, Associate Editor
Feilim Mac Gabhann, Editor-in-Chief
PLOS Computational Biology

Dear editors,

We thank you and the referees for the helpful and constructive reviews of our manuscript "Constructing benchmark test sets for biological sequence analysis using independent set algorithms" (PCOMPBIOL-D-21-01725), and for the opportunity to submit a revised manuscript. Below are our responses to each of the four reviews. You also specifically asked us to "*better explain the meaning of "independent" data and justify the use of percent identity (PID) as opposed to an estimated phylogeny to define the split into training and test data*", and this is primarily in our response to review #2, especially to the second point in that section.

Review #1

> *p3, line 71. You may have views about reducing redundancy in the training set, too. It might be interesting to state them.*

We see this as an issue for a homology search method, not for construction of a benchmark. In constructing realistic benchmarks, we keep the original alignment of the training sequences and their redundancy; real input alignments will contain redundant sequences. Different homology search methods will have their own ways of dealing with redundancy in input query alignments, and part of the benchmark is seeing how well they work. For most profile construction methods including HMMER's hmmsearch, for example, sequences are weighted so that common sequences are not overrepresented.

On line 83, we added a comment to state "(We preserve the alignment of the training set, including sequence redundancy; the goal of the benchmark is to have realistic query sequence alignments, which do often include redundant sequences. Different homology search methods deal with sequence redundancy in different ways. Most profile construction methods use relative sequence weights, downweighting similar sequences.)".

Sean R. Eddy
Investigator

Ellmore C. Patterson Professor
Molecular & Cellular Biology, and Applied Mathematics
Harvard University, Biological Laboratories 1008A
16 Divinity Avenue, Cambridge, Massachusetts 02138
+1-617-496-6757 | seaneddy@fas.harvard.edu | **eddylab.org**

In our implementation, we also added an option to filter redundant sequences in the training set the same way we do for the test set.

> *p5, line 101. The present article presents a complete and unified investigation. In several of your algorithms, however, one could retain some randomness after ordering the candidate lists not by random permutation, but by degree class (small to large) and then permuting randomly within each class of degrees. The effect would be to prioritize vertices of lower degree in the test and training sets, i.e., to ensure inclusion of outliers wherever possible. I suggest future examination of this variant of your algorithms.*

> *p.9, line 135. The article makes a good case for the virtues of randomness, so the previous comment is intended entirely as a suggestion.*

> *p.14, line 214. It would be interesting to know if the ordering by degree changed the difficulty of the search.*

We agree with the intuition that prioritizing vertices of lower degree is preferable because it would ensure inclusion of outliers whenever possible. Fortunately, our randomized algorithms implicitly favor the selection of lower degree vertices; the probability a vertex is included is inversely proportional to its degree. For this reason, we expect that the suggested variant would not behave drastically differently. (It is an interesting idea!)

To highlight the point about including outliers, we've added the sentence on line 130 "The bias towards including low degree vertices, which correspond to "outlier" sequences, is desirable for creating benchmarks that include the most remote homologs."

> *p.10, Figure 1. The X-axis is unlabeled. The legend states that it is "Fraction of families successfully split", but adding the axis label would help the top label "Performance..." (which can be retained). I admire the authors' ingenuity in introducing several dimensions of information into the plot.*

Thank you, we corrected this.

> *p.14, Figure 4. PID = percent identity. I could not find the full acronym, which should be explicit.*

We have removed the acronym PID and instead state "percent identity."

p.18, line 257. Who benchmarks the benchmarks? The observation that the benchmark changes the difficulty of the test search (and possibly, therefore, the robustness of training) but preserves the relative performance of the algorithms is very reassuring.

Initially we were surprised that the different benchmarks substantially changed the difficulty of the homology search test, which is why we investigated possible causes and came to understand why the difficulty varied. We too were reassured when we found that the relative performance is preserved.

Review #2

…they note that BLUE and COBALT are strictly better than CLUSTER with respect to the first two tests (how many families are split and how accurate the subsequent bioinformatics test is using this split). They also note that INDEPENDENT SELECTION is more accurate than BLUE and COBALT, but since INDEPENDENT SELECTION is successful at splitting a much smaller number of families, this is not that important.

To clarify, we would not describe independent selection as "more accurate." Rather, we would say that independent selection produces a benchmark that is less challenging than the benchmarks produced by the other methods, which causes the homology search methods to look more accurate. To make this more clear, we have reworded a sentence on line 235 to read "The reported accuracy for hmmsearch is highest for the benchmark produced by Independent Selection, followed by the benchmarks produced by Cobalt, Blue, and then Cluster."

I have two questions about the whole approach that I think would merit some discussion. The first question has to do with whether using PID to define the test and training data really gets at "independence", which the authors have justified by noting that two sequences datasets can be non-independent due to shared evolutionary history. Therefore, if the test data form a small clade in the evolutionary tree, separated by a fairly long branch from the training data, they would have perhaps good separation in terms of PID but would not be independent. Wouldn't a better approach be to construct a phylogeny based on the alignment, and then extract two clades from the phylogeny?

[that is:] *(1) Is PID a very good proxy for evolutionary independence, and why not instead use an estimated phylogeny to define the split into training and test data?*

Sequences related by phylogeny are never statistically independent. For benchmarking remote homology search methods, our goal instead is for the training and test sets to be **dissimilar**.

For benchmarking purposes, the advantages of a simple definition of dissimilar training and test sets (no pair better than x% identity) outweigh more sophisticated and accurate inference of tree topology and branch lengths. Moreover, splitting based on tree topology alone does not satisfy the goal of creating challenging, dissimilar training and test sets (all the sequences in both clades could still be quite similar to each other).

We made revisions to clarify. We added the following new second paragraph to the intro:

"We are specifically interested in benchmarking methods for remote sequence homology detection. We want to test how well a homology detection method, given a homologous clade of sequences as an input, can detect other homologous sequences in a distant outlying clade. We can create artificial cases of this by splitting known sequence families phylogenetically at deep ancestral nodes. The difficulty of detecting remote homologs depends more on the distance to the outlying sequences than on details of the tree topology. Therefore, inferring a complete tree topology is unnecessary; it is sufficient and more relevant to have a clear distance-based rule for establishing training and test set splits that are challengingly dissimilar."

We also added a sentence to the next paragraph, after describing single-linkage clustering: "This procedure is a fast proxy for building a phylogenetic tree from distances based on percent identity and selecting test sequences from the outlying clades.".

Where we described graph edges as representing a non-independent relationship, now (on line 94) we say edges represent similarity above threshold (as determined by percent identity). We still use the term independent set because this is the proper graph theory term for a set of vertices with no edges between them. An "independent set" does not correspond to sequences that are independent of each other; instead, an independent set corresponds to a set of sequences where no pair is sufficiently similar (as defined by the percent identity threshold).

> *The second question has to do with other aspects of defining training data and test data. An important consideration for training data is that it should be representative of the test data. It seems to me that by enforcing a large PID separation between test and training data in a sense is leading to the training data being very different from the test data. This may result in machine learning methods not generalizing well to the test data, since the test data are so different. This hypothesis is consistent with the results shown in the study, since their new methods end up producing splits into test and training data that have *smaller* gaps (in terms of PID) than the original CLUSTER method, and this is used to explain why the new methods have better accuracy. Wouldn't this also potentially suggest that relaxing the required gap between testing and training data even more might further improve accuracy in the subsequent bioinformatics analyses?*
>
> [that is:] *(2) Can the authors discuss the competing objectives in defining the training and test datasets, and characterize the different approaches in terms of these competing objectives?*

First we would note that the nature of this problem is that the training data should *not* be representative of the test data (i.e. not drawn from the same distribution); that is a main point of the work, and why standard machine learning benchmarking procedures are insufficient. The fundamental challenge of remote homology search is designing a method that learns the key features of the family and can generalize to identify remote members that are greatly diverged from the training sequences. To test remote homology search methods, the test data should be chosen to be remotely homologous to the training data. The new paragraph added to the introduction should help clarify this.

The percent identity threshold p is an important choice that should be decided based on the goals of the methods being benchmarked. By setting the percent identity threshold at 25%, we are effectively asking how well each homology search method can identify previously unseen homologs that are at most 25% identical to any training sequence. If we were to relax the percent identity threshold, we would absolutely expect the reported accuracy of the methods on the resultant benchmark to increase.

We leave the percent identity threshold as an input parameter so that the user can pick the best threshold for their application. For example, if a researcher was interested in comparing the performance of less sensitive but ultra fast homology search methods (e.g. DIAMOND), it would be advisable to set a higher percent identity threshold. It doesn't make sense to benchmark the ability of methods to detect homologs at 25% identity if the methods are not designed to detect homologs that far diverged.

To clarify, we have added a sentence on line 87 to emphasize that the threshold should be chosen based on application. Moreover, we added a sentence at line 191 explaining our choice of p=25%.

> *Finally, there are a few places where the writing could be improved. Some of them are really minor (low-level writing issues), but others have to do with clarity.*
> *1. What are "deep alignments"? (See lines 54 and 59)*

Deep alignments are alignments that include many sequences. We have removed the word "deep" and describe this explicitly in each place.

> *2. The problem formulation (beginning of "Results") clearly depends on parameters p and q. Their selection should be discussed, and the problem formulation should note this dependency.*

We prefer to keep the algorithm descriptions in the beginning of the results in terms of parameters p and q since these are input parameters to our software. We have added a sentence on line 87 to emphasize that these thresholds should be chosen based on application. We state our choice of p and q at the beginning of the section "Performance Comparisons" (line 185) since this is the first subsection whose results depend on the choice of p and q.

> *Also, since they will remove sequences, the problem formulation is inadequate: they need to note something about removing sequences (perhaps not too many).*

To address this, we have added a paragraph at line 89: "In order to guarantee that no test sequence has p% pairwise identity to any training sequence, some sequences will end up in neither the training nor the test set. Our algorithms find training and test sets, and they are returned if they are larger than the user-specified minimum acceptable size for each."

> *Then also perhaps something about choosing between alternative splittings of the input: what are the criteria?*

There is no choice of alternative splittings if the algorithm is run once. As described in lines 169-174, we have two options for running the algorithms multiple times: (i) the "run-until-n" option in which the algorithm runs at most n times and returns the first split that satisfies a user defined threshold, and (ii) the "best-of-n" option in which the algorithm runs n times and returns the split that maximizes the product of the training and test set sizes (i.e. the geometric mean).

3. The choice of what set becomes the training set and which one becomes the test set is not really discussed, beyond saying (lines 34 and 35) "one cluster (usually the largest one) becomes the training set". Is that wise? Isn't there a possibility that the larger one might be the easier or the harder one? Wouldn't picking the set that has the largest amount of heterogeneity provide a better training set?

The goal is to benchmark on realistic input alignments, and the larger cluster is more like the original input (Pfam) alignment, with a smaller number of sequences removed. We chose the larger cluster to be the training set to ensure that there are ample training sequences available. We also imagine that the larger set is more representative of the Pfam family. To clarify our reason we added the sentence (on line 136) “We assign the larger set as the training set because the goal is to benchmark on realistic input alignments, and the larger cluster is more like the original input alignment; additionally, we aim to benchmark methods that may require large numbers of training sequences.”

4. Lines 189-195. This is where the evaluation process is described. It would merit an additional sentence, e.g., "Here we test the ability of HMMER to detect whether a sequence contains a substring that is homologous to a family in PFAM" or perhaps something else? That way the "true negatives" and "true positives" are understood.

Agreed. At line 224 we have modified a sentence to read: “To study this, we used the four algorithms in a previously published benchmark procedure (Eddy11) that evaluates a method's ability to detect whether a sequence contains a subsequence homologous to a Pfam family.”

Low-level Writing:
Line 67. "Given set" -> "Given a set".
Line 143. Unless British English conventions are used, add a comma after "i.e."
Lines 144-145. By definition, a connected component is a maximal subset that is connected, therefore by definition there cannot be any edge between two connected components. This sentence should be rephrased. For example, replace "partitioned into connected components, such that" by "partitioned into connected components; by definition,"

Agreed; we made each of these changes.

Review #3

> *My main comment is that the motivation, and meaning of "independent" data, needs better explanation. The usual idea of machine learning is that same-class objects are nearby in some high-dimensional space, for some definition of nearby. Does that make them non-independent?*

It does not make them non-independent; you can still think of same-class objects that are nearby in some high-dimensional space as independently drawn from some distribution that is heavily concentrated on a specific region of the high-dimensional space. Rather, the key idea in many machine learning applications is that the data points x can be considered to be sampled i.i.d. (independent and identically distributed) from one distribution P(x), whatever that distribution is; that all samples are i.i.d. justifies randomly splitting known examples into training and test sets.

In contrast, biological sequences related by a phylogenetic tree are not independent, and a clade of known sequences is not identically distributed relative to a clade of remote homologs we seek to detect.

To clarify this point we have modified the sentence on line 21 to say: "In many areas of machine learning and statistical inference, data samples can be thought of as approximately independent samples from some distribution describing the data, and in this case a standard approach is to randomly split available data into a training and a test set." The new second paragraph should also help clarify the point, and the final paragraph of the Discussion is also relevant.

> *For example, a previous paper ("Benchmarking the next generation of homology inference tools" Saripella et al. 2016) constructed a benchmark "aiming to ensure... homologies at different evolutionary distance". Perhaps that is more representative/realistic, than ensuring distant homologies only?*

Our benchmark is specifically focused on evaluating the ability of methods to detect remote homologs that are <p% identical to known members of the family. By changing the threshold p, we can construct easier or harder benchmarks and get a detailed sense of which method does best at different percent identity ranges. Since homology search methods tend to do well at high percent identity, we chose to focus on a benchmarking the difficult case (< 25% identity). The newly added second paragraph in the intro should help clarify.

Additionally, a key difference between Saripella et al. and our approach is that our splitting algorithms give a training set and a test, whereas Saripella et al. identifies

pairs of homologous sequences. In order to benchmark profile methods (such as HMMER and PSI-BLAST) we need an aligned training set from which to build a profile.

> *In the present paper, the final Discussion paragraph states that homology search is an "out of distribution" task, but that is not obviously true. For example, if we learn from mammal sequences then try to find bacterial sequences, that is indeed out-of-distribution, but why can't we learn from diverse sequences and thereby be "within distribution"?*

Identifying homologs *within* a clade of known sequences (diverse or not) is typically not challenging for current methods. In the newly added second paragraph, we clarify that we are specifically focused on benchmarking **remote** homology search; finding distantly related sequences is what makes it out-of-distribution.

> *"Out of distribution" sounds like "transfer learning": may or may not be worth mentioning.*

Out of distribution and transfer learning are different concepts. Transfer learning refers to pre-training a model on a general task (typically, where we have lots of training data), and then adjusting the parameters of this model by training on a different, usually more specific, task (typically, where we have only limited data).

> *The "Cluster" method uses one (largest) connected component as the training set. This seems obviously pessimal, because it tends to minimize the diversity of the training set. Could this be why the Cluster benchmark is harder?*

If the largest connected component did not have much diversity, it seems plausible that this would make the benchmark harder. However, it is not clear whether or not the largest cluster will be lacking in diversity generally. One reason the Cluster algorithm fails is that the largest connected component contains all or almost all of the sequences. It can be the case that the largest cluster contains a diverse set of sequences and the test set contains a small number of outliers.

> *Not critical, but it would be interesting to see the runtime breakdown into the two steps.*

We did not explicitly test this, but we are confident that the runtime is dominated by the first step. In the second step, there are far fewer sequences to consider and so the algorithm runs much faster.

The homology benchmark has a severe and easily-fixable problem: the negative sequences are shuffled thus lack low-complexity/simple repeats, which are a significant issue in practice. Easy fix: use reversed instead of shuffled sequences. I realize this is somewhat tangential to the paper's main point.

We agree that the benchmarks described here do not exercise a major failure mode of sequence homology searches on low-complexity/simple repeat sequence, and we also agree it's tangential; it's also complicated and would take space to explain. Reversed sequences are not safe to use in benchmarks this way, contrary to intuition and some use in the literature. Counterintuitively, a sequence is more likely to have a higher alignment score to its reverse than to a random sequence, and thus reversed sequences generate spurious false positives. This is even true for i.i.d random sequences and their reverses! The issue arises from palindrome occurrence statistics (briefly: for any given random k-mer, it is more likely to find a palindromic k-mer match at random in the same sequence, thus creating an alignment of 2k matches between the sequence and its reverse, than it is to find 2k matches between two random sequences.)

It would be helpful to briefly summarize what is known about the "well-studied" graph problem of independent sets. Maybe mention maximal versus maximum. Finding a maximum set (or even approximating it?) is theoretically hard. Are randomized algorithms known to be advantageous?

Thank you for the suggestion. We have elaborated beginning on line 112, "While it is NP-hard to find a maximum size independent set in a graph [14], randomized algorithms can be applied to quickly find a maximal independent set (an independent set where if any additional vertex were added, the set would no longer be an independent set). The Blue and Cobalt methods are inspired by two such algorithms [15,16]. Unlike Cluster, Blue and Cobalt always find a maximal independent set in task(ii)."

Review #4

Comment 1: If an input set is split into two dissimilar sets, it seems likely that these sets will correspond to distinct functionally divergent subgroups. If so, then a protein model obtained using the training set will fail to correspond to the test set: i.e., one will be comparing apples and oranges. In that case a search program might be justified in failing to detect a test sequence given that it belongs to a different subgroup.

We have added a new second paragraph in the Introduction to clarify that the problem we are focused on is remote homology search. It is definitely true that remote homologs may not have similar functions (or even similar structures, in rare cases!) but it is still of interest to successfully detect them.

> *Of course, what the authors appear to want is a model of the superfamily features upon which one could also superimpose a mixture of the family and subfamily models within that superfamily. Hence, to detect a distant member of the superfamily that corresponds to a subgroup or an orphan sequence absent from the training set, one should perhaps only model the superfamily features. This is often done by purging closely related sequences from the input set so as to retain only the most highly conserved residue positions as well as the more subtle residue propensities at other positions corresponding to the characteristics and the structural coe of the superfamily. Alternatively, one could retain all sequences, but down weight them for sequence redundancy rather than purging the set.*

The work is about constructing appropriate benchmarking training/test sets for evaluating different remote homology search methods. We are agnostic to the approach taken by different homology search methods. We agree with the reviewer that some methods purge closely related sequences, and some methods use relative and/or absolute sequence weights.

> *Comment 2: Although purging the input set of all but the most diverse sequences seems like a good way to avoid training biases, this can lead to undesirable side effects. For example, input sets often include pseudogene products and sequences corresponding to DNA open reading frames containing frame shifts or splicing artifacts. Since such sequences will lack similarity to functional sequences, they will be overrepresented in the purged set. Hence, focusing too much on the most diverse sequences can, of course, add a significant amount of misleading noise to the training set. Hence, to better capture the key features of both the superfamily and of each subgroup within the superfamily it might make more sense to first group sequences into closely-related clusters (each, to some degree, corresponding to a functionally divergent subgroup) and then select, as a representative for each cluster, that sequence sharing the greatest similarity, on average, with the other sequences in the cluster.*

We agree; see comment above. This is about what a homology search method does with its input sequence alignment, and not really about how one constructs a training/test split for benchmarking.

Comment 3: Some protein families are very highly conserved across distantly related organisms. For example, histone H4 from pea and cow differ at only 2 out of the 102 positions. This makes it easy, of course, to distinguish these proteins from unrelated proteins. However, distinguishing such highly conserved protein family members from other related proteins may not be trivial. I assume, of course, that this is not the problem that the authors are seeking to address, even though it too is an important problem when seeking to assign specific functions to hypothetical proteins. I suggest that the authors describe very precisely the nature of the problem that their new programs aim to address. This appears to be the problem of identifying very distant homologs to the more typical members of a protein superfamily...

The new second paragraph clarifies that we are focused on the problem of remote sequence homology detection.

Comment 4: I suggest that the authors may get a better perspective on these questions by generating simulated sequences from a known distribution (e.g., based on an HMM) and then adding additional sequences by simulating their evolution, which would model the lack of statistical independence that their programs aim to address.

The nature of the remote homology search problem – that the sequences we aim to recognize are in a distant clade, relative to the available training sequences – makes this a nontrivial simulation to attempt. Realistically simulating sequences down a tree with a *homogeneous* evolutionary model with insertion/deletion is already challenging; remote clades tend not to be following the same evolutionary rates as other clades on the tree, and developing a method of simulating remote clades of evolved sequences in a realistic way is a difficult task that we believe is beyond the scope of this paper.

Minor comments:
1. In Figure 3, what do the gray bars above the colored bars that fade out at their top ends indicate?

These are the scatter points of the outliers. Each point is somewhat translucent so that it is possible to see the density of outliers. We added "as bar plots including outliers (translucent grey circles" to the Figure legend.

2. Page 15 line 209: "brought closer the ROC curve" --> "brought closer to the ROC curve"?

Agreed; corrected.

Again, we thank you and the reviewers for your comments, and we hope our revised manuscript is viewed favorably!

Sincerely,

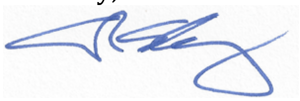

Sean R. Eddy

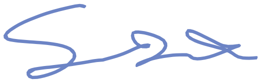

Samantha N. Petti
