# Supplementary material for: Constructing benchmark test sets for biological sequence analysis using independent set algorithms
Source: PLoS Comput Biol. 2022 Mar 7;18(3):e1009492. doi: 10.1371/journal.pcbi.1009492 (PMC8929697; doi:10.1371/journal.pcbi.1009492)
Supplement: Attachment — Submitted filename: response-to-reviews-v2_2_8_22.docx [file pcbi.1009492.s005.docx]

8 February 2022

Maricel G Kann, Associate Editor

Feilim Mac Gabhann, Editor-in-Chief

PLOS Computational Biology

Dear editors,

We thank you and the referees for the helpful follow-up reviews of our manuscript “Constructing benchmark test sets for biological sequence analysis using independent set algorithms” (PCOMPBIOL-D-21-01725). Below are our responses to reviewer 3.

**Review #3**

*Sorry if I wasn't clear enough last time. I still feel that much of
the writing about "independent" data is confusing, ambiguous, and
misleading. I think there are two different reasons for this.

Reason (1): Independence is relative, not absolute. According to the
mathematical definition, two things A and B are independent when:
P(A|B) = P(A).
But this depends on the "background" probability distribution, P(A).
Consider these two background probability distributions for sequences:

(i) Random i.i.d. sequences, with some length distribution.

(ii) Randomly pick from a set of real biological sequences, which are
related by evolution.

It is possible for sequences to be independent relative to (ii) but
not (i).

Reason (2): While the title and abstract do not specify "homolgy
search" (and indeed the methods may be useful for other things, like
protein localization prediction), the paper focuses on the aim of
homology search. This is confusing, because "homology" means "related
by evolution", which is the same as the non-independence "nuisance"
that they are trying to eliminate!

In short, I feel that the abstract and introduction should be
carefully rewritten to clarify these issues. Surely the authors
understand all this, and it's well-described in the final paragraph of
Discussion. But the Abstract and Introduction are too unclear.*

We agree that the abstract was vague when it described training and test sets for typical machine learning applications as “independent.” We removed this description entirely and do not mention the notion of independence until the introduction where we have space to be precise about what we mean by the term. Our abstract is now focused on the specific challenge of splitting sequence data so that the training and test sets are dissimilar.

Now the first mention of independence begins on line 35, when we state that “In many areas of machine learning and statistical inference, data samples can be thought of as approximately independent samples from some unknown distribution describing the data.” When we introduce the problem of remote homology search on line 49, we now use the precise language previously in the discussion “The remote homologs $y$ are not from the same distribution as the known sequences $x$; they are drawn from some different distribution $P(y \mid x,t)$, where $x$ are the known sequences and $t$ accounts for evolutionary distances separating remote homolog $y$ from the known

examples $x$ on a phylogenetic tree.”

*The paper should at least mention the issue of low-complexity/simple
repeats, and that it might affect the relative performances of the
homology search methods. (I suspect DIAMOND may have an advantage in
avoiding simple repeats.)*

We agree that performance of different homology search software packages depends on how they handle simple repeats and biased composition regions, and indeed on many other issues too. But in this work we are not trying to dissect why one method outperforms another; rather, we are showing that the relative order of performance for different tools is robust to different choices of clustering method. We feel that a discussion of why one tool might be doing better than another one is tangential to the point of our paper.

*Page 20: should "the known sequence x" be "the known sequences x"?

Page 20 last line: x and y mixed up?*

Thank you. We have corrected these issues.

Again, we thank you and the reviewers for your comments.

Sincerely,

Sean R. Eddy

Samantha N. Petti
